# Spatiotemporal Variation in Marine Mammal Antipredator Behaviors Resulting From a Predation Pinch Point

**DOI:** 10.1002/ece3.72841

**Published:** 2026-02-02

**Authors:** Conner M. Hale, Joffrey Jouma'a, Astarte Brown, Patrick W. Robinson, Rachel R. Holser, Daniel P. Costa, Roxanne S. Beltran

**Affiliations:** ^1^ Department of Ecology and Evolutionary Biology University of California Santa Cruz California USA; ^2^ Institute of Marine Sciences University of California Santa Cruz California USA

**Keywords:** biologging, *Mirounga angustirostris*, predation, risk

## Abstract

Predator–prey relationships are fundamental aspects of ecological systems that determine the behavior and distribution of animals across time and space. Variation in predation risk can be used to explore when and why individuals perform antipredator behaviors. In marine environments, it is difficult to observe predation and antipredator behaviors. Fortunately, biologgers have long been used to study the distribution and dive behaviors of northern elephant seals ( *Mirounga angustirostris* ) on their twice‐yearly foraging trips. Here, we analyzed the horizontal and vertical movements of 353 adult female seals across 17 years to investigate how they move through a “predation pinch point”—an area where predators and prey co‐occur due to habitat features. Specifically, we explored the diel timing of departure from and arrival to the colony and spatial concentrations in benthic diving (diving close to/along the seafloor). Benthic diving and temporal concentrations in movement to and from the colony may serve antipredator functions, such as minimizing detection and ambush by predators. We found that only the timing of departure from and not arrival to the colony showed any significant temporal pattern. Seals tended to depart during the late afternoon or at night but arrived throughout the day. Spatially, there were consistent patterns of benthic dives during the first and final parts of their trips as seals crossed the continental shelf. By combining dive, location, bathymetry, and predator data, we were able to identify how seals modified behaviors that likely help them avoid predators. These findings illustrate how animals respond to varying levels of predation risk and can be used to develop more precise dynamic landscapes of fear.

## Graphical abstract

Using 17 years of biologging data from 353 adult female northern elephant seals, we explored how seals modified their use of time and space when moving through nearshore areas where predation risk is high. We found that seals tended to depart at night, arrive during the day, and perform benthic dives along the continental shelf. These behaviors likely reduced the likelihood of seals being detected by predators, and help us understand how animals respond to varying levels of risk in their environment.

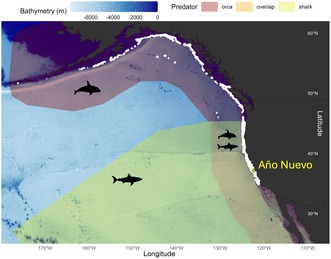

## 1 Introduction

Predator–prey relationships are fundamental aspects of ecological systems that determine the behavior and distribution of animals across time and space. Predators exert consumptive (e.g., mortality) and nonconsumptive (e.g., behavioral changes) effects on prey (Lind and Cresswell 2005; Nelson et al. 2004; Relyea 2003). Nonconsumptive effects include changes to migration timing and space use in prey (Furey et al. 2016; Moxley et al. 2020; Newsome et al. 2017; Sabal et al. 2021; Sortland et al. 2024; Wirsing et al. 2008). These effects are especially detectable at “predation pinch points”—areas where predators and prey co‐occur due to habitat features that concentrate their movement (Heithaus et al. 2009; Quinn et al. 2017; Sortland et al. 2024). Antipredator behaviors, such as vigilance and habitat selection (Clark 1994; Ives and Dobson 1987), can increase fitness by preventing mortality. However, these behaviors can also lead to increased energetic requirements (e.g., through avoidance maneuvers) or prevent necessary behaviors (e.g., foraging) which can reduce fitness (Brown and Godin 2023; Lind and Cresswell 2005). Consequently, individuals performing antipredator behaviors must assess the level of perceived risk and respond appropriately (Ives and Dobson 1987; Lima and Bednekoff 1999). The resulting “dynamic landscapes of fear” explain that prey distributions change across spatiotemporal scales in response to perceived predation risk (Gaynor et al. 2019; Laundré et al. 2010, 2001) and predictable changes in the natural world (Beltran et al. 2021; Palmer et al. 2022). This framework helps answer questions about the function of different behavioral strategies across risk landscapes, but in many prey species, the timing, characteristics, and prevalence of antipredator behaviors are not well understood.

Direct predation and resulting antipredator behaviors are well‐documented in terrestrial ecosystems (Laundré et al. 2010, 2001), but are more difficult to observe in the ocean. This presents a critical knowledge gap as behavioral effects resulting from predation risk are believed to be more pronounced in aquatic environments (Preisser et al. 2005). Fortunately, biologgers—instruments attached to animals that can measure location, intrinsic variables, and environmental data (Beltran et al. 2025; Williams et al. 2020)—make it possible to determine how marine animals change their behaviors in response to one another in the open ocean (Block et al. 2011; Jorgensen et al. 2019; Klimley et al. 2001; Weng et al. 2007). Biologging studies have revealed critical information about habitat use (Andrzejaczek, Chapple, et al. 2022; De Vos et al. 2015; Domeier et al. 2012; Sequeira et al. 2018) and the spatiotemporal timing of antipredator behaviors (Aguilar De Soto et al. 2020; Arnould and Hindell 2001; Beltran et al. 2021; Morse et al. 2019) in marine environments.

Adult female northern elephant seals ( *Mirounga angustirostris* ) are a model species for biologging studies because they are abundant, relatively easy to instrument, and exhibit site fidelity (Costa et al. 2024; Lowry et al. 2014). Known predators of northern elephant seals include white sharks ( *Carcharodon carcharias* ) and transient orcas ( *Orcinus orca* ) (Ainley et al. 1981; Baird and Dill 1996; Klimley 1994; McInnes, Lester, et al. 2024; McInnes, Trites, et al. 2024). White sharks are visual predators that concentrate hunting activity during crepuscular periods (Johnson et al. 2009; Martin et al. 2005; Martin and Hammerschlag 2012). White sharks approach prey from below, enabling them to detect and ambush prey that are silhouetted by light from the sun or moon (Fallows et al. 2016; Huveneers et al. 2015). Transient orcas specialize in marine mammals and prey on elephant seals as they cross the continental shelf (Baird and Dill 1996, 1995; McInnes, Lester, et al. 2024). As visual hunters, transient orcas reduce their use of echolocation as a strategy against prey with acute underwater hearing abilities (Barrett‐Lennard et al. 1996; Deecke et al. 2005; Ford et al. 1998; Riesch and Deecke 2011).

Spatially heterogeneous landscape features can influence predator–prey interactions by affecting prey vulnerability and perceived risk (Gorini et al. 2012), habitat use (Gotceitas and Colgan 1989; Heithaus et al. 2009), and the use of antipredator behaviors (Gaynor et al. 2019). They can also affect predator hunting strategies (Heithaus et al. 2009) and their efficiency (Crowder and Cooper 1982). In the ocean, physical environmental features (e.g., bathymetry) have been used to explain animal movement and behavior (Braun et al. 2022; McConnell et al. 1992). Here, we explore how features associated with a “predation pinch point” (i.e., bathymetry and the distance to Año Nuevo) affect the occurrence and prevalence of at‐sea antipredator behaviors in adult female northern elephant seals.

While at sea, elephant seals spend most of their time below the surface (Robinson et al. 2012). Examining their dives, documented using time‐depth recorders, can provide insight into their at‐sea behaviors. Most dives by adult female northern elephant seals are to 400–600 m deep (Costa et al. 2024). Dives exhibited by northern elephant seals vary in their shape and functionality, falling into categories that include: transit (Crocker et al. 1994), foraging (Asaga et al. 1994), drift (Mitani et al. 2010; Robinson et al. 2010), and benthic dives (Hassrick et al. 2007; Le Boeuf et al. 1993). As air‐breathing marine mammals, elephant seals must balance their need to surface for air with the predation risks associated with the photic zone, where their predators are most abundant (Andrzejaczek, Lucas, et al. 2022; McInnes, Lester, et al. 2024). In adult female northern elephant seals, mass, but not age, influences dive depth and duration (Favilla and Costa 2020; Hassrick et al. 2010), despite age and mass being correlated (Le Boeuf 1994). Deep diving has been suggested to serve a predator avoidance function for many large marine vertebrates (Braun et al. 2022), including northern elephant seals (Crocker et al. 1994; Le Boeuf and Crocker 1996).

We asked when, where, why, and how adult female northern elephant seals modify (i) diving behavior and (ii) departure/arrival timing at the start and end of their twice‐yearly foraging trips. Because elephant seals overlap in space and time with coastal predators (e.g., white sharks and orcas, Andrzejaczek et al. 2025; Jorgensen et al. 2019; McInnes, Lester, et al. 2024) during these nearshore windows, we integrated > 10 years of dive and location data with predator distribution and abundance data to test the prediction that seals shift diel timing and reduce benthic use in predator‐rich coastal zones. We further tested whether season (post‐breeding vs. post‐molt), trip phase (departure vs. arrival), and individual state (age, mass) explain variation in these responses; we did not expect age or size to mediate decisions strongly.

## 2 Materials and Methods

### 2.1 Study Site and Animal Handling

We instrumented 353 female northern elephant seals ( *Mirounga angustirostris* ) between the ages of 3 and 14 (median: 6) at Año Nuevo Reserve (CA, USA 37.11 N, 122.34 W). Each seal was instrumented with a combination of Argos (*n* = 275) or FastLoc GPS plus Argos (*n* = 78) satellite transmitters and time‐depth‐light recorders (Wildlife Computers SPOT [Argos] and MK10 [Argos + GPS], Sea Mammal Research Unit CTD and CTD/F [Argos]). Argos instruments have a higher degree of spatial error than GPS instruments (Costa et al. 2010), but variation in data quality was accounted for during data pre‐processing steps (see below). Instruments were deployed between 2004 and 2020 during the post‐molt (*n* = 142) and post‐breeding (*n* = 211) foraging trips. The post‐molt (~June to January) trip lasts around 8 months and the post‐breeding (~February to May) trip lasts around 2 months (Robinson et al. 2012). Details about animal handling can be found in Costa et al. (2024). Our dataset contains a total of 2,624,221 dives (1,484,185 foraging, 873,168 transit, 219,167 drift, and 47,701 benthic) across all years and seals.

### 2.2 Data Preparation

Costa et al. (2024) described the data collection, initial calibration, and quality control procedures for tracking and diving data, whereas the data processing, including dive type classification, behavioral modeling, and predator‐risk analyses, were conducted for the present study.

#### 2.2.1 Track Data Preprocessing

Animal tracks were processed following the methods described in Costa et al. (2024). Briefly, location data were obtained from either the Argos satellite tracking system or both the Argos and Fastloc‐GPS tracking systems, depending on the instrument used. Typically, we received 8 to 10 Argos location estimates per 24‐h period (Costa et al. 2024). Using the *aniMotum* R package (Jonsen et al. 2023), we fitted a correlated random walk (maximum travel rate of 3 m per second) to our location data to re‐estimate the animal's position every 3 h. This allowed us to account for variability in location quality and obtain location estimates at regular time steps at a similar scale to the lower‐quality (Argos only) raw location data. Because elephant seal foraging trips are at a much larger scale (~3300 km maximum distance) than location error (Robinson et al. 2012) and typically follow an East–West direction which is not associated with location bias (Costa et al. 2010), we do not expect that these variable location qualities had a substantial impact on the conclusions presented here.

#### 2.2.2 Time‐Depth Recorder Data Preprocessing

Diving data were processed and quality controlled as described in Costa et al. (2024). Depth data were collected at sampling intervals ranging from 1 to 8 s and were downsampled to 8 s to facilitate comparison across individuals. Zero‐offset correction, dive identification, and dive analysis were achieved using the IKNOS (Y. Tremblay; Costa et al. 2024) toolbox in MATLAB (The MathWorks Inc.). Dives were identified as excursions from the surface reaching a minimum depth of at least 25 m and lasting at least 32 s (Costa et al. 2024). Dives were classified into one of four types based on shape that reflects approximate function (benthic, transit, foraging, or drift) using a program designed to detect the unique characteristics of each dive type (e.g., depth, duration, ascent and descent rates, shape) recorded by time‐depth recorders (Hassrick et al. 2007; Jouma'a et al. 2024; Kienle et al. 2022; Le Boeuf et al. 1988; Robinson et al. 2012, 2010).

#### 2.2.3 Bathymetry Information

Bathymetric data with a resolution of four arc minutes were retrieved from the ETOPO 2022 database hosted on the National Oceanic and Atmospheric Administration website using the *marmap* R package (Pante and Simon‐Bouhet 2013) to help identify benthic dives. Specifically, we used the *getNOAA.bathy* function and the following specifications: lon1 = −180, lon2 = −105, lat1 = 60, lat2 = 25, resolution = 4. We changed positive bathymetry values (i.e., above the ocean surface) to zero, and then converted the remaining raw bathymetric data (negative values beneath the ocean surface) to positive values to match the diving depth data. We then assigned a bathymetry measurement to each dive based on the latitude/longitude of that dive from the interpolated movement tracks (interpolation is described in Costa et al. 2024).

### 2.3 Dive Type Classification

#### 2.3.1 Foraging

Foraging dives were identified based on an index, created by Robinson (Robinson 2009), that identifies and scores putative foraging dives. Because elephant seals exhibit a variety of diving behaviors during the bottom phase of presumed foraging dives, most parameters inferred from time‐depth profiles (e.g., foraging depth, size, and frequency of vertical excursions [“wiggles”]) can vary substantially. The “Foraging intensity index” was designed to assess foraging dives that encapsulate several key traits associated with activity, scaled to the size of the bottom phase:

$$\text{Foraging intensity index}=W\times V/R+\left[\left(W\times V/R\right)\times \left(V/T\right)\right]$$

where *W* = number of “wiggles” during the bottom phase, *V* = total vertical distance traveled during the bottom phase, *R* = range of depth values during the bottom phase, and *T* = duration of the bottom phase.

Dives that exhibit many “wiggles” during the bottom phase (i.e., a Foraging intensity index greater than 35) were considered foraging dives. The foraging‐dive classification index uses depth (m) and time (s) as input variables and applies an empirically derived threshold of 35, developed from manually validated Año Nuevo dive records (Robinson 2009). While this automated approach has been widely used to ensure consistency across large datasets (e.g., Robinson et al. 2012; Naito et al. 2013), we note that formal quantitative validation against manual classifications has not yet been published and should be undertaken in a future study.

#### 2.3.2 Drift

Drift dives were identified based on the first derivative (vertical component of velocity) of the time‐depth profile following methods described in Robinson et al. (2010). For each dive, a kernel density estimation of the vertical speed was used to find both the drift rate (position of the peak) and the relative proportion of the dive spent drifting at the dominant drift rate (height of the peak). If the height of the peak exceeded a density of one, a large proportion of the dive was done at the dominant drift rate and thus considered a drift dive (Jouma'a et al. 2024).

#### 2.3.3 Benthic

Dives were considered benthic if they (i) were not classified as foraging or drift dives; (ii) had square corners; (iii) had a bottom phase slope close to zero; and (iv) if the difference between the maximum depth of the dive and the bathymetry was less than 100 m. This conservative bathymetry threshold accommodates spatial uncertainty in both bathymetry (~7.4 km resolution) and location estimates. To characterize the bottom phase, we calculated a kernel density of bottom phase vertical speed for each dive. Following Jouma'a et al. (2024), if the peak fell within the range of ±0.08 m per second (nearly flat bottom) and the height of the peak exceeded a density of 1.5 (consistent vertical speed), it was classified as a benthic dive. Additionally, best‐fit lines were drawn for the descent phase and the bottom phase, and the intersection between the two lines was checked against the animal's actual trajectory. If the actual trajectory was less than 15 m from the intersection point (i.e., the switch from descent phase to bottom phase was sharp), it was identified as a benthic dive.

#### 2.3.4 Transit

Dives that did not meet any of the above identification criteria were classified as putative transit dives. This terminology reflects that, while their shape and dive‐phase characteristics match the profiles previously identified as transit dives in manually validated datasets, true horizontal displacement cannot be confirmed without 3‐dimensional tracking data.

#### 2.3.5 Caveats

Due to substantial variation in the characteristics of dives, some dives were undoubtedly misclassified. However, due to the large number of dives in our dataset, we choose to utilize this objective but imperfect method rather than subjectively classifying each dive. A decision tree illustrating how dives were classified is available in the Supporting Information (Figure S1).

### 2.4 Predator Abundance and Distribution Data

To interpret the behaviors we observed in our dataset, we used information from three published studies (Andrzejaczek et al. 2025; Jorgensen et al. 2019; McInnes, Lester, et al. 2024) that described the spatial and temporal distribution of white sharks and transient orcas in central California. Jorgensen et al. (2019) used 7 years of electronic tagging data from white sharks in the northeastern Pacific and 26 years of orca observational survey data from the Southeast Farallon Islands to understand how competition between the two predators affected their abundance and distribution at foraging sites (pinniped rookeries) between 2007 and 2013. They found that white shark occurrence peaked in fall (October–November), that orca occurrence peaked in spring (May), and that these predators only co‐occurred during fall (September–November; Jorgensen et al. 2019). Andrzejaczek et al. (2025) used 15 years (2006–2022) of telemetry data to describe habitat use for 327 white sharks in central California. They found that the coastal phase of white sharks extended from mid‐August to mid‐February, and that Año Nuevo was a main aggregation site (Andrzejaczek et al. 2025). McInnes, Lester, et al. (2024) used 15 years (2006–2021) of observational data to describe the foraging behavior, diet, seasonal occurrence, and habitat use patterns of transient orca in and around Monterey Bay. They found that orca occurrence was highest in April and May, that observations of orcas were highest along the continental shelf break, that orca pods used prey‐specific foraging tactics, and that adult female northern elephant seals were preyed upon when returning to Año Nuevo (McInnes, Lester, et al. 2024).

### 2.5 Data Analyses

#### 2.5.1 Diving Behavior

**FIGURE 1:**
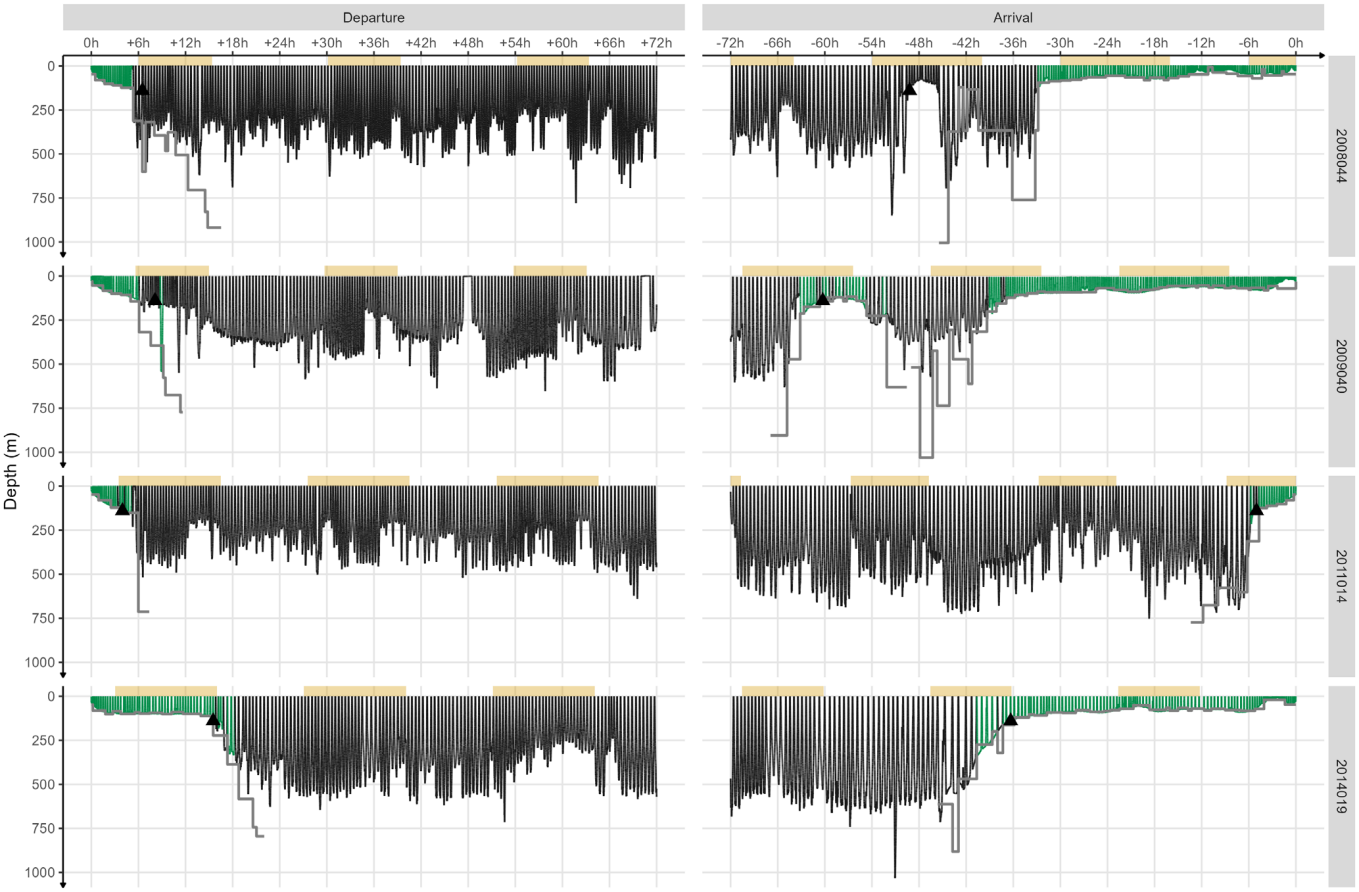
Snapshot of departure and arrival dive profiles for four randomly selected representative adult female northern elephant seals during their post‐molt (individuals 2008044, 2009040) trips or post‐breeding (individuals 2011014, 2014019) trips. Individual IDs are labeled in the facets. Departure and arrival were constrained to 72 h to maintain individual dive visibility. Shading indicates time of day (yellow = daylight), coloration indicates dive type (green = benthic, black = other), and gray lines represent the bathymetry depth for each seal. The black triangles indicate the temporal transition from on‐shelf to off‐shelf (for departure) and off‐shelf to on‐shelf (for arrival).

**FIGURE 2:**
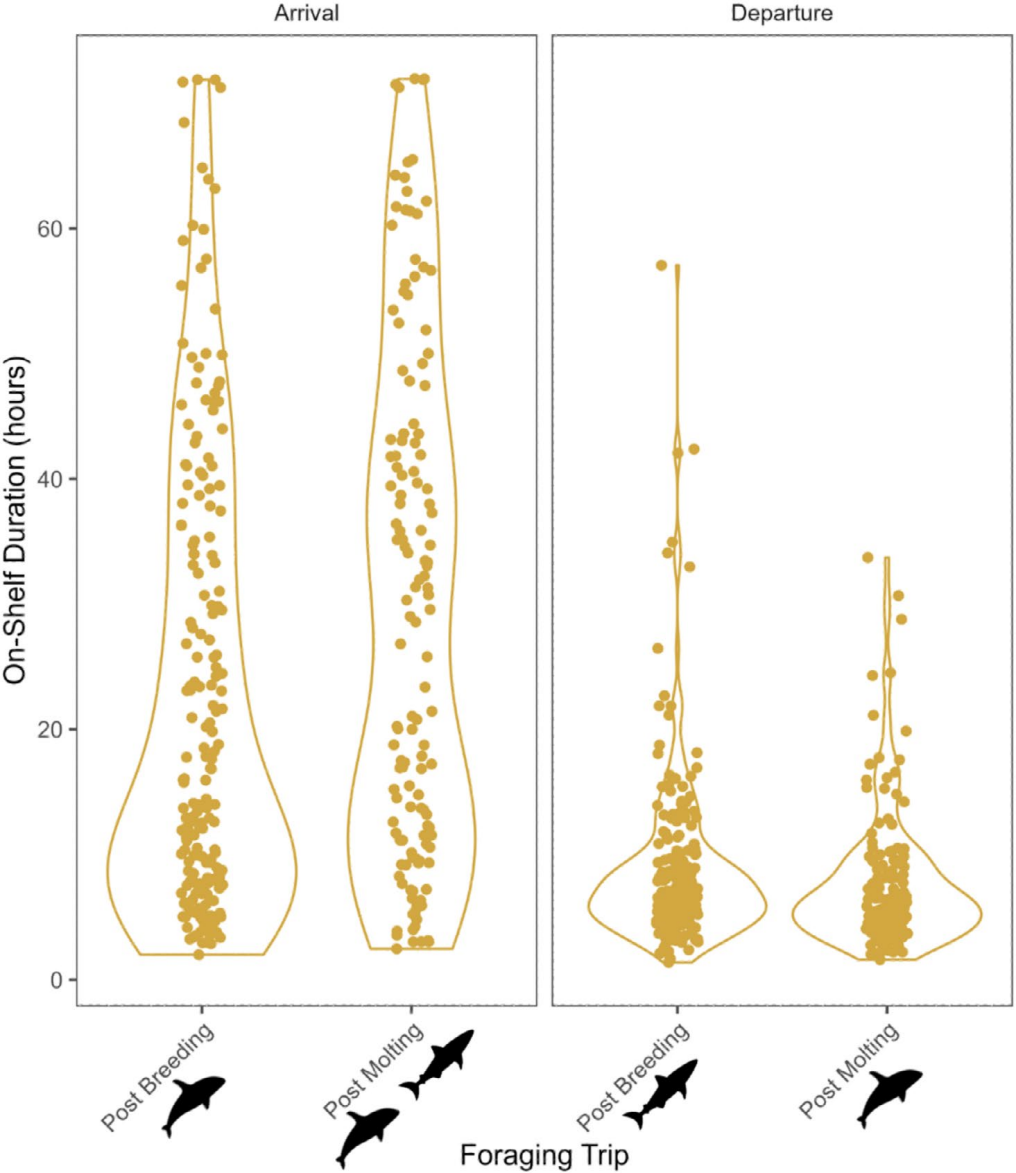
Adult female northern elephant seals (*n* = 340) spent significantly more time on the shelf when arriving than when departing. Time spent on the shelf was more variable for arrival than departure and was lowest during departure for the post‐molt foraging trip. Predator icons denote their general seasonal occurrence in the Año Nuevo State Marine Reserve and Monterey Bay areas (per Andrzejaczek et al. 2025; Jorgensen et al. 2019; McInnes, Lester, et al. 2024).

**FIGURE 3:**
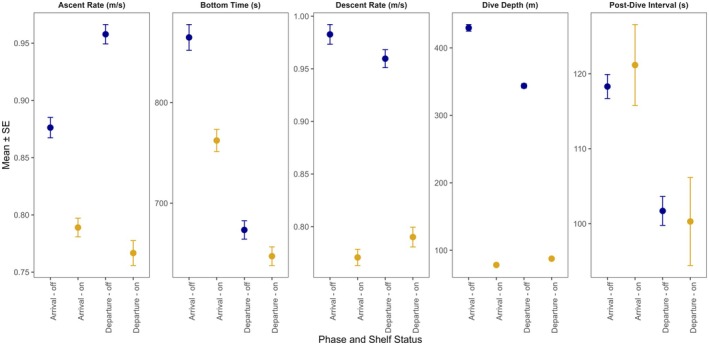
Mean ± SE dive metrics of adult female northern elephant seals (*n* = 340) by trip phase (arrival/departure) and shelf status (on/off continental shelf). Data points and error bars are grand means and standard errors of each seal's mean metric across all dives for that trip phase and shelf status. Ascent rates were highest when seals were off the continental shelf; bottom times were higher during arrival than departure; descent rates were higher when seals were off the shelf; dive depths were higher off the shelf; and post‐dive intervals were longer during arrival than departure.

We used binomial generalized linear models (GLMs) to test how body mass, age, and season (post‐breeding vs. post‐molt) influenced the proportion of benthic dives overall and within predator areas (Table 1). Because individual ID explained very little variation in the models, we opted to use GLMs without random effects. Due to the coastal concentration of white sharks (Andrzejaczek et al. 2025; Klimley et al. 2001) and transient orcas (Jorgensen et al. 2019; McInnes, Lester, et al. 2024) while hunting, we also analyzed the horizontal and vertical movement metrics of elephant seals relative to on‐ and off‐shelf areas. This allowed us to determine if seal behaviors changed on and off the shelf, as a proxy for predation risk. Following prior research, we defined the continental shelf break as occurring at 140 m bathymetric depths (Greene et al. 2002; Le Boeuf and Crocker 1996). To estimate the duration of continental shelf use by individual seals, we analyzed time‐depth records spanning the first and last 72 h of each trip, corresponding to departure and arrival phases, respectively (Figure 1). Shelf use was defined by clusters of five consecutive benthic dives that met the maximum depth criteria of ≤ 140 m. For the departure phase, shelf use duration was measured as the time between the first and last qualifying dives before the first sustained block of deeper (off‐shelf) dives. For the arrival phase, shelf use duration was calculated as the time span of all subsequent shallow benthic dives beginning with the first qualifying block that followed an extended period of deeper (off‐shelf) diving. To identify these transitions, we applied run‐length encoding to each seal's dive sequence within each phase. For each qualifying block, we recorded the transition time (on‐shelf → off‐shelf for departure; off‐shelf → on‐shelf for arrival) as the timestamp of the first dive in the run and calculated the total elapsed time between the first and last dive within the identified on‐shelf sequence. For each seal and trip phase (departure or arrival), we summarized dive behavior during on‐ and off‐shelf periods by extracting dives around the shelf crossing (Figures 2 and 3). We used Gamma (link = “log”) GLMMs to quantify (i) how on‐shelf durations varied with trip phase and the effect of season after accounting for individual random effects (Figure 2) and (ii) how dive ascent rate, bottom time, dive descent rate, dive depth, and post‐dive intervals varied with trip phase and shelf status after accounting for individual random effects (Figure 3). The models were set up with interactions among predictors, and we dropped the 2‐ or 3‐way interactions if they were not significant. The contribution of each predictor to the model was assessed using coefficient and *p*‐value estimates. To account for the strong physical influence of dive depth on metrics such as ascent rate, descent rate, bottom time, and post‐dive interval, we fit these models with mean dive depth (Depthcov) as a continuous covariate (Table 2). This approach allowed us to assess whether behavioral differences among seasons, phases, and shelf statuses persisted after controlling for variation in dive depth. We validated all models before interpretation to ensure assumptions were met. We examined residual plots to assess homoscedasticity and influential points, calculated variance inflation factors to check for multicollinearity among predictors, and inspected Q–Q plots for normality of residuals. Variance inflation factor (VIF) values were generally low to moderate for most predictors. However, the inclusion of mean dive depth (Depthcov) as a covariate substantially increased collinearity with shelf status, which is expected because bathymetry and depth are inherently correlated.

**TABLE 1:** Summary of the frequency of benthic and all dive types (benthic, drift, forage, and transit) for the post‐breeding (*n* = 211) and post‐molt (*n* = 142) foraging trips in adult female northern elephant seals. “W/o location” refers to diving data that do not have paired location data. The total proportion of benthic dives that occurred throughout the trips relative to all other dive types is displayed, as is the proportion of benthic dives that occurred specifically within predator habitats (see Figure 6).

	# Seals	All dives		Benthic dives		% Benthic dives per area			
		Total	W/o location	Total	Proportion	Shark	Orca	Total	Overlap
Post‐breeding	211	956,309	8	27,446	2.9%	80.3%	100%	100%	80.3%
Post‐molt	142	1,667,912	0	20,255	1.2%	83.2%	97%	97%	83.2%
Overall	353	2,624,221	8	47,701	1.8%	82.2%	98.1%	98.1%	82.2%

**TABLE 2:** Summary of generalized linear (GLM) model results for dive behavior metrics across elephant seal foraging trips (* = interactive, + = additive). Significant values (*p* ≤ 0.05) are bolded.

Model	Formula	Term	Estimate	SE	*t*/*z* value	*p*
GLM1	Ascent Rate ~ Depthcov + trip * phase * shelf_status	(Intercept)	−0.3270010	0.0155252	−21.063	**< 2E‐16**
		Depthcov	0.0009684	0.0001012	9.566	**< 2E‐16**
		tripPost Molting	0.0333712	0.0211245	1.580	1.14E‐01
		phaseDeparture	−0.0905985	0.0190821	−4.748	**2.28E‐06**
		shelf_statusoff	−0.2010577	0.0431280	−4.662	**3.45E‐06**
		tripPost Molting:phaseDeparture	0.1213400	0.0298227	4.069	**5.00E‐05**
		tripPost Molting:shelf_statusoff	−0.0903852	0.0308130	−2.933	**3.41E‐03**
		phaseDeparture:shelf_statusoff	0.1994604	0.0295334	6.754	**2.14E‐11**
		tripPost Molting:phaseDeparture:shelf_statusoff	0.0343726	0.0425981	0.807	4.20E‐01
GLM2	Bottom Time ~ Depthcov + trip * phase * shelf_status	(Intercept)	6.5494200	0.0211597	309.523	**< 2E‐16**
		Depthcov	0.0003875	0.0001380	2.808	**0.005053**
		tripPost Molting	0.1322600	0.0287912	4.594	**4.76E‐06**
		phaseDeparture	−0.0863121	0.0260076	−3.319	**9.29E‐04**
		shelf_statusoff	0.0265490	0.0587803	0.452	6.52E‐01
		tripPost Molting:phaseDeparture	−0.1938351	0.0406462	−4.769	**2.06E‐06**
		tripPost Molting:shelf_statusoff	−0.0839688	0.0419959	−1.999	**0.045761**
		phaseDeparture:shelf_statusoff	−0.0800666	0.0402519	−1.989	**0.046889**
		tripPost Molting:phaseDeparture:shelf_statusoff	0.0697235	0.0580581	1.201	2.30E‐01
GLM3	Descent Rate ~ Depthcov + trip * phase * shelf_status	(Intercept)	−0.03074	0.01366	−22.505	**< 2E‐16**
		Depthcov	0.001082	0.00008905	12.154	**< 2E‐16**
		tripPost Molting	−0.09606	0.01858	−5.169	**2.70E‐07**
		phaseDeparture	−0.05151	0.01679	−3.069	**0.00219**
		shelf_statusoff	−0.12260	0.03794	−3.232	**0.00126**
		tripPost Molting:phaseDeparture	0.16210	0.02623	6.178	**8.61E‐10**
		tripPost Molting:shelf_statusoff	−0.05209	0.02711	−1.922	5.49E‐02
		phaseDeparture:shelf_statusoff	0.04191	0.02598	1.613	0.10692
		tripPost Molting:phaseDeparture:shelf_statusoff	0.04355	0.03747	1.162	0.24531
GLM4	PDI ~ Depthcov + trip * phase * shelf_status	(Intercept)	4.8776596	0.0567741	85.913	**< 2E‐16**
		Depthcov	−0.0005939	0.0003702	−1.604	0.10890
		tripPost Molting	−0.0906812	0.0772504	−1.174	0.24066
		phaseDeparture	−0.2192405	0.0697815	−3.142	**1.72E‐03**
		shelf_statusoff	0.1064230	0.1577149	0.675	0.49993
		tripPost Molting:phaseDeparture	0.0893581	0.1090588	0.819	0.41273
		tripPost Molting:shelf_statusoff	0.1979811	0.1126803	1.757	0.07914
		phaseDeparture:shelf_statusoff	0.1233074	0.1080009	1.142	0.25377
		tripPost Molting:phaseDeparture:shelf_statusoff	−0.3550776	0.1557771	−2.279	**0.02280**
GLM5	Dive Depth ~ trip * phase * shelf_status	(Intercept)	4.33078	0.01208	358.501	**< 2E‐16**
		tripPost Molting	0.06732	0.01892	3.559	**3.86E‐04**
		phaseDeparture	0.14152	0.01706	8.294	**2.64E‐16**
		shelf_statusoff	1.79636	0.01708	105.149	**< 2E‐16**
		tripPost Molting:phaseDeparture	−0.06859	0.02671	−2.568	**0.010341**
		tripPost Molting:shelf_statusoff	−0.23218	0.02675	−8.678	**< 2E‐16**
		phaseDeparture:shelf_statusoff	−0.41064	0.02413	−17.017	**< 2E‐16**
		tripPost Molting:phaseDeparture:shelf_statusoff	0.18838	0.03778	4.987	**6.95E‐07**
GLM6	shelf_duration_hr ~ tag_type + trip + phase	(Intercept)	2.86569	0.09207	31.126	**< 2E‐16**
		tag_typeArgos	0.14193	0.09361	1.516	0.129
		tripPost Molting	0.10042	0.07988	1.257	0.209
		phaseDeparture	−0.99975	0.04398	−22.731	**< 2E‐16**
GLM7	pct_benthic ~ mass + trip	(Intercept)	−4.07011	0.36694	−11.092	**< 2E‐16**
		I(DepartCalcMass/1000)	0.44406	1.14329	0.388	0.698
		tripPost Molting	−0.75194	0.09681	−7.767	**8.03E‐15**
GLM8	pct_benthic_orca ~ mass + trip	(Intercept)	−4.06258	0.36709	−11.067	**< 2E‐16**
		I(DepartCalcMass/1000)	0.41884	1.14376	0.366	0.714
		tripPost Molting	−0.78489	0.09688	−8.102	**5.41E‐16**
GLM9	pct_benthic_shark ~ mass + trip	(Intercept)	−4.09620	0.32043	−12.784	**< 2E‐16**
		I(DepartCalcMass/1000)	0.31691	0.99841	0.317	0.751
		tripPost Molting	−0.79854	0.08458	−9.442	**< 2E‐16**

#### 2.5.2 Temporal Distribution

**FIGURE 4:**
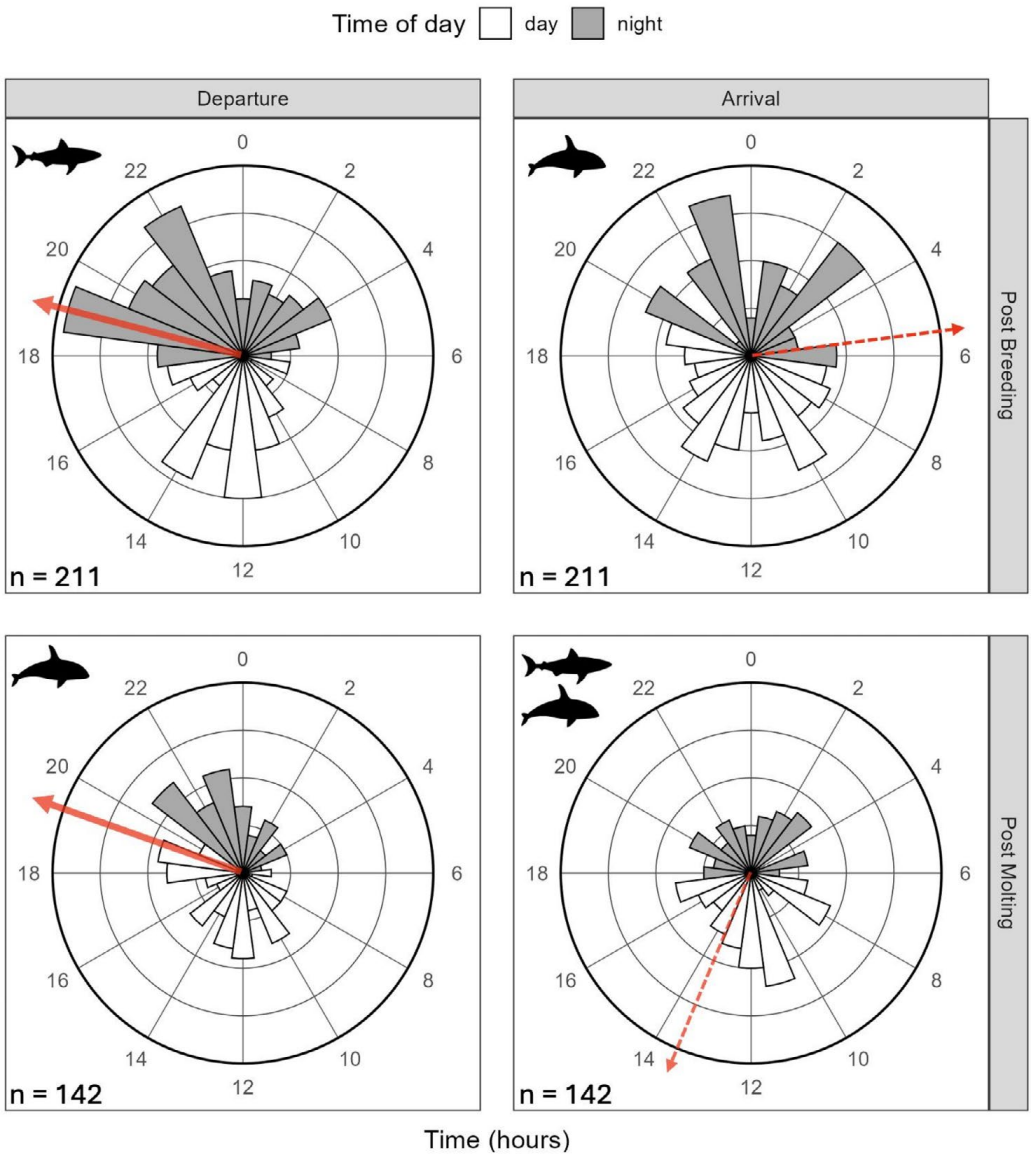
Circular distributions of the first (i.e., departure) and last (i.e., arrival) dives of adult female northern elephant seals (*n* = 353) for their post‐breeding and post‐molt foraging trips. Variation in dive timing was present in all phase‐season pairs but was lower for departure than arrival. Shading indicates local night and non‐shading indicates local day relative to the Año Nuevo colony, California (UTC +7), inner‐concentric rings represent counts of occurrence (5, 10, 15 seals), predator icons indicate general seasonal occurrence in the Año Nuevo State Reserve and Monterey Bay areas (per Andrzejaczek et al. 2025; Jorgensen et al. 2019; McInnes, Lester, et al. 2024), and red arrows indicate the average timing of departure and arrival (solid = significant, dashed = nonsignificant).

We defined day and night as follows: if the start of a dive took place between local sunrise and the subsequent sunset, it was assigned to the daytime, whereas if the start occurred between sunset and the following sunrise, it was assigned to the nighttime. Sunset and sunrise were calculated based on the location of each dive using the *suncalc* R package (Thieurmel and Elmarhraoui 2022), in which a sunrise is defined as the moment when the top edge of the sun appears on the horizon and a sunset when the top edge of the sun disappears below the horizon. To examine temporal variation in the timing of departure and arrival across foraging trips (Figure 4), we calculated the average hourly timing of occurrence for the first (i.e., departure) and last (i.e., arrival) dive for each seal. To assess the significance of the mean resultant vector length (*R̄*) of departure (i.e., the first dive) and arrival (i.e., the last dive) timing, we performed a Rayleigh test of uniformity using the *CircStats* R package (Lund and Agostinelli 2018). An *R̄* value closer to one indicates that the data show strong deviation from circular uniformity (Moxley et al. 2020).

#### 2.5.3 Spatial Distribution

**FIGURE 5:**
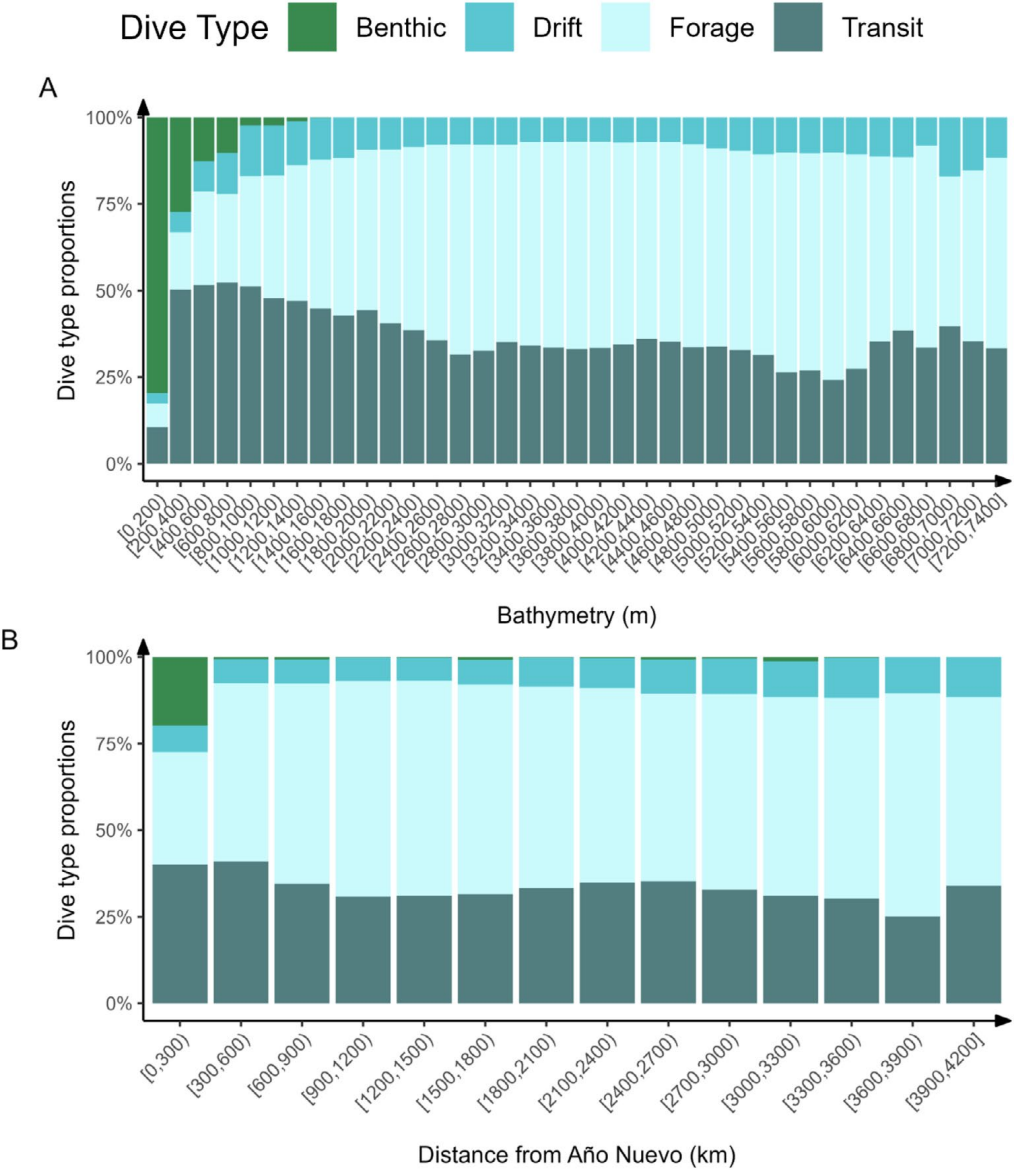
The proportion of benthic dives performed by adult female northern elephant seals (*n* = 353) decreased as a function of bathymetry (A) and distance from the Año Nuevo colony (B). Proportions of other dive types (i.e., drift, transit, and foraging) increased as seals moved further from Año Nuevo into the bathymetrically unrestricted open ocean. *X*‐axes show arbitrary bins (200 m for Bathymetry, 300 km for Distance). Bracket notation follows standard conventions: ( = exclusive lower bound and ] = inclusive upper bound.

To determine how the proportions of dive types exhibited by seals changed across their foraging trips (Figure 5), distance from the Año Nuevo colony was estimated for each dive location (i.e., using interpolated track locations) and seal using the *distGeo* function from the *geosphere* R package (Hijmans 2022).

## 3 Results

### 3.1 Data Summary

From the 353 adult female northern elephant seals, we recorded 47,701 benthic dives across all years and trips (Table 1, Table S1). Benthic dives accounted for an average of 1.8% of all dives across all individuals and trips (Table 1; PB: 2.9% and PM: 1.2%). Departure for the post‐breeding trip, summarized across all study years, occurred in February (February 16th ± 9 days) and arrival occurred in late April/early May (May 1st ± 14 days). Departure for the post‐molt trip, summarized across study years, occurred in late May/early June (June 6th ± 9 days) and arrival occurred in late December/January (January 12th ± 22 days) (see Beltran et al. 2024).

### 3.2 Diving Behavior

We found that the duration seals spent on the continental shelf was significantly shorter during departure than during arrival, regardless of season (Figure 2). Ascent and descent rates were strongly influenced by shelf status, with both being faster off‐shelf than on‐shelf (Figure 3). After accounting for mean dive depth, these relationships remained significant but were reduced in magnitude, confirming that some of the raw differences were due to depth‐related physical constraints rather than strictly behavioral adjustments. Depth itself was a strong predictor of ascent rates, descent rates, and bottom time (*p* < 0.01 for all), but season‐ and phase‐level effects persisted, and a phase‐dependent shelf effect remained for ascent rate, with off‐shelf ascents particularly rapid during departure (Figure 3). Bottom time did not differ between on‐ and off‐shelf dives during departure but was longer off‐shelf during arrival, even after controlling for depth (Figure 3). Post‐dive interval duration was not significantly affected by depth or shelf status but remained shorter during departure than arrival (Figure 3). Season also influenced several metrics: bottom times were longer, descents were faster, and dives were deeper during the post‐molt trip (Table 2). Ascent rates and post‐dive intervals were unaffected by season, while the number of benthic dives was significantly higher during the post‐molt trip, including within shark and orca areas (Table 2). Age did not contribute significantly to any relationships after accounting for mass.

### 3.3 Temporal Patterns

Seals showed variability in the timing of departure and arrival across both foraging trips (Figure 4). For both trips, departure showed a weak but significant concentration around 19:00 h, which corresponded to either night or soon before (Figure 4; PB; 19.4 h, PM; 19.7 h). Although departure timing was varied and occurred during almost all hours of the day, there was a significant trend for both trips (Figure 4; PB; *R̄* = 0.2, *p* < 0.001, PM; *R̄* = 0.17, *p* = 0.02). Arrival timing was non‐significant and occurred during almost all hours of the day (Figure 4; PB; *R̄* = 0.01, *p* = 0.97, PM; *R̄* = 0.1, *p* = 0.26). Arrival from the post‐breeding trip tended to occur before sunrise (Figure 4; PB; 5.7 h), and arrival from the post‐molt trip tended to occur during the daytime (Figure 4; PM; 13.7 h).

### 3.4 Spatial Patterns

**FIGURE 6:**
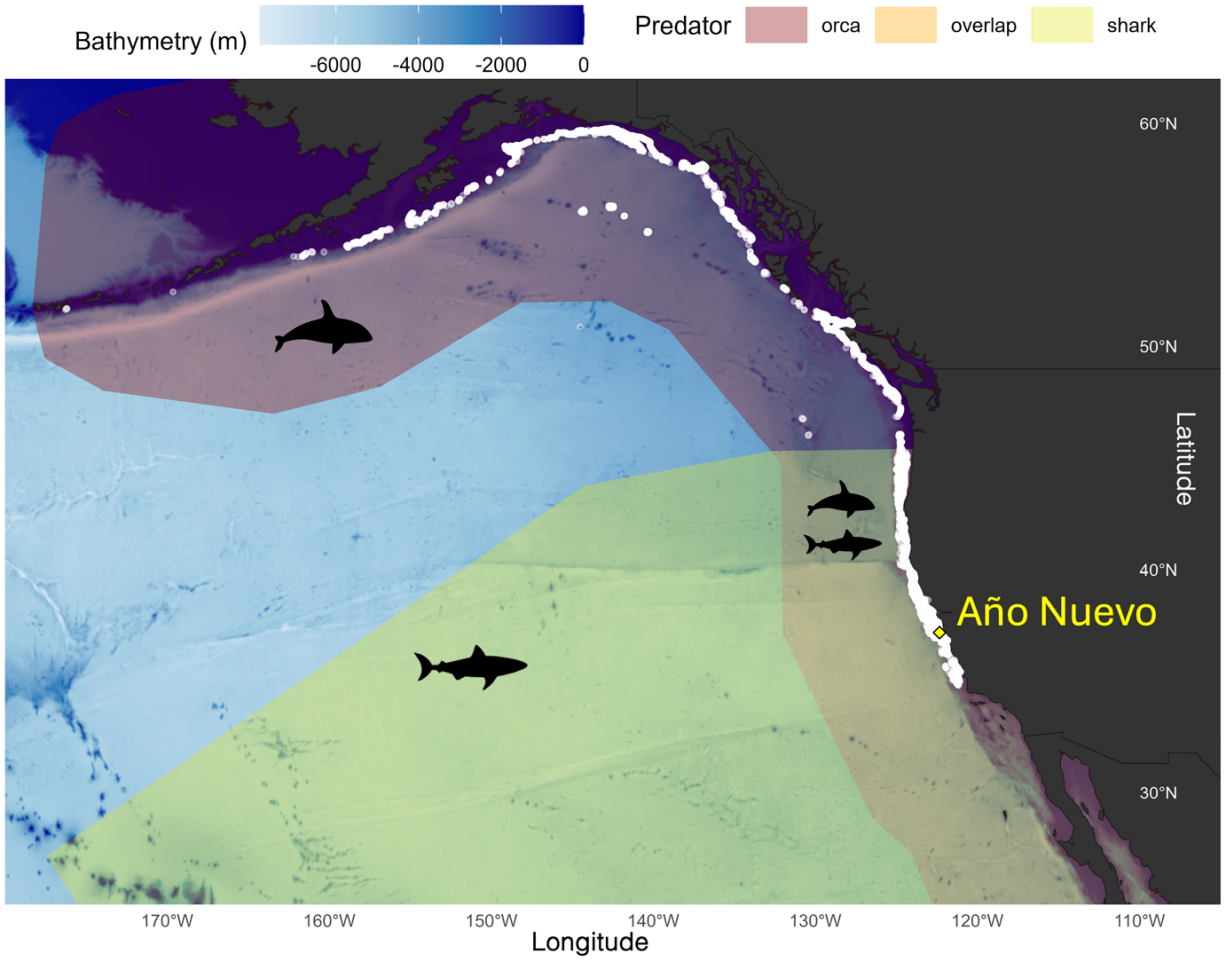
Map displaying the spatial overlap of benthic dives (white dots) performed by adult female northern elephant seals (*n* = 353) within known predator areas: orca (red polygon), shark (yellow polygon), and predator overlapping (orange polygon) areas (adapted from Jorgensen et al. 2019). The Año Nuevo colony is represented by the yellow diamond.

Benthic dives were most highly concentrated in the area around Año Nuevo but were present along the coast throughout the animals' range (Figure 6). Benthic dives occurred as far as ~3600 km away from Año Nuevo, but were limited to areas where bathymetry did not exceed 1600 m (Figures 5 and 6). The proportion of dive types measured across the post‐breeding and post‐molt foraging trips varied with ocean bathymetry (Figure 5A) and distance from the Año Nuevo colony (Figure 5B). As seals moved farther from the colony, into deeper waters, the proportion of benthic dives recorded decreased (Figure 5). Results were consistent across tag types: patterns in benthic dive proportion (Figure 5) and spatial distribution (Figure 6) were qualitatively similar between Argos and Argos + GPS datasets, and tag type did not significantly influence the on‐shelf duration (Table 2), indicating that differences in spatial accuracy between tag types did not alter the main ecological relationships.

## 4 Discussion

By combining dive, location, bathymetry, and predator data, we were able to investigate when, where, why, and how adult female northern elephant seals modified their behaviors in areas and during periods of high predation risk. Our data revealed patterns in the diel timing of departure, a lack of patterns in the diel timing of arrival, and that benthic diving was limited to bathymetrically shallow areas of the coast. Additionally, we found that season and mass, but not age, contributed to the patterns observed. Previous research on adult female northern elephant seals suggested that, when on the shelf, seals would (i) depart or arrive at night, (ii) swim along the sea floor when departing and arriving, and (iii) descend rapidly to minimize time spent near the surface where visual predators are most active, then ascend more slowly once below the primary attack zone. These behaviors can be interpreted as strategies to reduce detection and attack by predators (Le Boeuf and Crocker 1996). The substantial dataset analyzed here allows us to follow up on these questions, which were posed nearly 30 years ago, and contribute to literature addressing how animals mediate risk across dynamic landscapes of fear.

### 4.1 Temporal Patterns

As suggested previously (Le Boeuf and Crocker 1996), seals tended to depart from the Año Nuevo colony at night. However, we did not observe any significant pattern with respect to the timing of arrival. By strategically timing their departure, seals may be able to reduce their interactions with predators during periods of their trips in which the continental shelf imposes a depth constraint on their diving abilities and when light conditions make them more conspicuous.

For both trips, the average hourly timing of departure occurred during 19:00 h (Figure 4). During departure for the post‐breeding trip, seals likely left at night because it reduces their likelihood of being preyed upon. Not only are white sharks less likely to be actively hunting at these times (Huveneers et al. 2015; Martin and Hammerschlag 2012), but departing at night could reduce the likelihood of sharks spotting seal silhouettes (Tricas and McCosker 1984). Departure for the post‐molt trip occurred during the final hours of daylight (Figure 4). Though departing during daytime may make seals more vulnerable to detection from visual predators, seals may use other tactics, such as adjusting spatial dive characteristics (e.g., ascent rates, bottom time), to avoid predators. Although departure timing showed statistically significant directionality, the low mean vector lengths (*R̄* < 0.2) indicate weak clustering around the mean, suggesting that departure decisions are flexible and likely influenced by multiple interacting factors (e.g., weather, light conditions, and local visibility) rather than a single driver.

Arrival timing varied for both trips such that there were no significant patterns (Figure 4). On average, seals arrived from the post‐breeding trip before sunrise, while arrivals from the post‐molt trip occurred during daytime (Figure 4). Arrival from the post‐breeding trip (April–May) occurs during a period of high orca abundance and low shark abundance, and arrival from the post‐molt trip (December–January) occurs during a period of high shark and orca abundance (Figure 2; Andrzejaczek et al. 2025; Jorgensen et al. 2019; McInnes, Lester, et al. 2024). During arrival, seals may rely more on their ability to modify spatial dive characteristics than on their ability to modify their timing, even though daytime sunlight conditions may increase the risk of being detected by predators (Beltran et al. 2021; Huveneers et al. 2015). Previous research on Australian fur seals ( *Arctocephalus pusillus doriferus* ) found similar concentrations in the timing of departure but not arrival (Arnould and Hindell 2001).

The lack of a pattern in the diel timing of arrival may arise for two reasons. The northern elephant seal breeding and molting seasons represent the largest aggregations of adult female seals each year (Le Boeuf and Laws 1994) and begin shortly after adult female seals return to Año Nuevo from their post‐molt and post‐breeding foraging trips. Although arrival for the breeding season is highly seasonally synchronous among adult female northern elephant seals (Beltran et al. 2024; Condit et al. 2022), diel synchrony is harder to achieve. While slowing down to ensure that they arrive within a specific time frame (e.g., at night) could reduce detection risk, it would extend the amount of time spent in nearshore waters where predation risk is higher than on land (Spraker et al. 2014). In other words, seals may accept the short‐term risk associated with moving during the day to minimize the time spent within the “predation pinch point.” Additionally, intra‐specific competition or predation (Pyle et al. 1999) between white sharks and orcas can reduce predation rates for seals (Jorgensen et al. 2019), which may cause mismatches in predation risk that result in a lack of antipredator behaviors (Gaynor et al. 2019).

### 4.2 Spatial Patterns

Our results suggest that adult female northern elephant seals perform spatially concentrated benthic dives on their foraging trips. In bathymetrically restricted areas of the coast where predators are more common, seals are likely using their deep diving abilities to remain close to the bottom of the seafloor to reduce predation risk (Le Boeuf and Crocker 1996). The exploitation of the spatially heterogeneous bathymetry associated with the “predation pinch point” near the Año Nuevo colony and the use of antipredator behaviors can reduce the likelihood of predator–prey encounter rates and may prevent the predation sequence (Suraci et al. 2022) from reaching the attack or kill stage. Diving along the seafloor, instead of at the surface or within the water column, may reduce ambush predators' (Black et al. 2023; Martin and Hammerschlag 2012) directions of attack. Although the resolution of the bathymetry data is somewhat coarse and may lead to occasional discrepancies between benthic dive depths and seafloor depth estimates, the overall spatial patterns remain robust. In fact, elephant seal dive data can provide valuable fine‐scale bathymetric information that complements and enhances coarse global datasets (McMahon et al. 2023).

As seals moved farther from the colony into deeper waters, the proportion of dive types they performed shifted (Figure 5). In the open ocean, adult female northern elephant seals use extreme physiological and behavioral adaptations, including increased oxygen stores (Favilla and Costa 2020) and limited sleep requirements (Kendall‐Bar et al. 2023), to spend most of their time feeding on prey at depths (> 200 m) where predators are scarce (Biuw et al. 2003; Hassrick et al. 2010; Le Boeuf et al. 2000; Miller et al. 2010; Andrzejaczek, Lucas, et al. 2022). Seals have previously been shown to modulate resting behaviors by sleeping at depth during the day to reduce their likelihood of being preyed upon (Beltran et al. 2021; Kendall‐Bar et al. 2023; Le Boeuf et al. 1988; Mitani et al. 2010).

Although most benthic dives occurred on the shelf break, some took place in areas off the shelf where the bathymetry was shallow enough to allow benthic diving (Figure 6). These dives were likely flat‐bottom shaped foraging dives taking place at seamounts and/or on the continental slope. Though less common than typical pelagic foraging, there are records of female seals foraging atop seamounts (Maxwell et al. 2012) and along the continental shelf (Adachi et al. 2021; Kienle et al. 2022). Foraging atop seamounts, like those in the Kodiak‐Bowie chain and the Mendocino Ridge (Hayes et al. 2013), may allow females to forage in highly productive benthic regions that are lower risk than areas on the shelf. Thus, while benthic dives near the colony may serve both predator‐avoidance and opportunistic foraging functions, benthic dives at offshore seamounts and along the continental slope are more likely to reflect primarily foraging activity. Because our data cannot directly distinguish these behavioral motivations, we interpret these patterns as context‐dependent rather than mutually exclusive functions.

Limitations on dive depth imposed by the continental shelf may be counteracted by seals using other behavioral modifications to minimize predation risk. For example, seals may adjust their movement rates in shallow, higher‐risk environments, possibly to balance vigilance with navigation or prey‐searching behaviors. Only for the on‐shelf departure and off‐shelf arrival phases did we find that descent rates were higher than ascent rates, which is counter to expectation (Figure 3; Le Boeuf and Crocker 1996). Descent rates being lower than ascent rates during the on‐shelf arrival phase may be further support for the argument that seals are prioritizing getting to the beaches of Año Nuevo as quickly as possible. Because dive depth exerts a strong biomechanical and physiological influence on dive shape, we explicitly incorporated mean dive depth as a covariate in our models to separate physical constraints from behavioral modulation. As expected, depth accounted for much of the variation in ascent rates, descent rates, and bottom time, but the persistence of phase‐dependent differences (particularly for ascent rates, Table 2) suggests that seals still modify behavior across trip phases and shelf environments beyond what can be explained by depth alone. These models confirm that seals exhibit consistent and predictable changes in dive behavior when entering and exiting high‐risk areas, suggesting that predation risk structures spatial and vertical movement patterns. Ultimately, successful departure and arrival are critical as seals need to maximize time at sea spent foraging (Beltran et al. 2023) and maintain their annual cycle (Beltran et al. 2024).

The behavioral adjustments described here have also been observed in the congener species, southern elephant seals ( *M. leonina* ). Male and female elephant seals display sexual dimorphism and sex‐specific foraging strategies that are driven by their reproductive strategies and energetic needs (Hindell et al. 2021; Kienle et al. 2022; Le Boeuf et al. 2000; Lewis et al. 2006) and are preyed upon by orcas (Condy et al. 1978; Hoelzel 1991; Reisinger et al. 2011). Foraging along the continental shelf enables seals to gain mass quickly but presents more predation risk than the open ocean, a trade‐off that males are more likely to make (Allegue et al. 2022; Kienle et al. 2022). Biologging studies on adult female southern elephant seals reveal that during the first and final parts of their foraging trips, seals perform deep dives believed to serve a predator avoidance function (Hindell, Slip, and Burton 1991; Slip et al. 1994). Time‐depth records also indicate that dive type shifts occur once seals initially cross the continental shelf (fig. 3 in Campagna et al. 1995), similar to those described here (Figure 1). Additionally, time spent foraging is limited while seals are on the shelf (Campagna et al. 1995; Hindell, Burton, and Slip 1991), likely to minimize the time spent in high‐risk areas. While moving through a “predation pinch point,” adult females of both species seem to modify their dive behaviors in a manner that serves a predator avoidance function.

Similar behavioral adjustments have been observed in other large marine vertebrates with the same predators. For example, Cuvier's ( *Ziphius cavirostris* ) and Blainville's beaked whales ( *Mesoplodon densirostris* ) perform silent ascents to impede the ability of orcas to track their movements (Aguilar De Soto et al. 2020). In addition to ascending silently, adult female northern elephant seals may also perform dive inversions—switches from ascent to descent that extend the dive duration—when they detect a predator. This behavior has been recorded in juvenile elephant seals in response to orca vocalizations (Fregosi et al. 2016; Shen 2023).

Our results support previous observations that marine prey species can behaviorally modify their movement in time and space as a predator avoidance strategy (Aguilar De Soto et al. 2020; Arnould and Hindell 2001; De Vos et al. 2015; Laroche et al. 2008; Morse et al. 2019; Moxley et al. 2020). The addition of a temporal characteristic to predation pressures transforms the landscape of fear to a dynamic landscape of fear and provides insight into how animals respond to predation risk in their multi‐dimensional ocean environments (Beltran et al. 2021; Gaynor et al. 2019; Laundré et al. 2010, 2001; Palmer et al. 2022). Important next steps include measuring the effectiveness of dive type and timing as an antipredator strategy. For example, the simultaneous deployment of biologgers on predators and prey could directly link antipredator behaviors and predation attempts. Additionally, biologging studies on juvenile northern elephant seals could demonstrate whether the behaviors described here are exhibited by younger, more naive individuals.

## 5 Conclusion

“Predation pinch points” exist within many ecological systems across time and space and can demonstrate how animals modify their use of habitat and behavioral strategies to avoid predation. Here, we show that adult female northern elephant seals modify their timing and their use of three‐dimensional space in areas of increased predation risk. This study contributes to the development of dynamic landscapes of fear in marine environments, where direct observations of predator–prey interactions are difficult.

## Author Contributions

**Conner M. Hale:** conceptualization (equal), formal analysis (supporting), investigation (equal), methodology (supporting), visualization (supporting), writing – original draft (lead), writing – review and editing (lead). **Joffrey Jouma'a:** conceptualization (equal), data curation (lead), formal analysis (lead), investigation (equal), methodology (lead), visualization (lead), writing – original draft (supporting), writing – review and editing (supporting). **Astarte Brown:** conceptualization (equal), formal analysis (supporting), investigation (equal), methodology (supporting), visualization (supporting), writing – original draft (supporting), writing – review and editing (supporting). **Patrick W. Robinson:** data curation (supporting), writing – review and editing (supporting). **Rachel R. Holser:** data curation (supporting), writing – review and editing (supporting). **Daniel P. Costa:** data curation (lead), funding acquisition (equal), writing – review and editing (supporting). **Roxanne S. Beltran:** conceptualization (equal), data curation (supporting), formal analysis (supporting), funding acquisition (equal), investigation (supporting), methodology (supporting), resources (lead), supervision (lead), visualization (supporting), writing – original draft (supporting), writing – review and editing (supporting).

## Funding

Full funding information for the dataset can be found in Costa et al. (Costa et al. 2024). Additional funding was provided by the Packard, Beckman, and Sloan Foundations, the Office of Naval Research, the National Science Foundation, the California Ocean Protection Council, and the Elysea Fund. Data were collected under National Marine Fisheries permits 786–1463, 87–143, 14636, 19108, and 23188, and approved by the University of California, Santa Cruz Institutional Animal Care and Use Committee (IACUC).

## Conflicts of Interest

The authors declare no conflicts of interest.

## Supporting information


**Figure S1:** ece372841‐sup‐0001‐FigureS1.docx.

**Table S1:** ece372841‐sup‐0002‐TableS1.xlsx.

## Data Availability

Data and code used here are available on Dryad https://doi.org/10.5061/dryad.mkkwh71bh.
