# Supplementary figures and images for: Spatiotemporal Variation in Marine Mammal Antipredator Behaviors Resulting From a Predation Pinch Point
Source: Ecol Evol. 2026 Feb 2;16(2):e72841. doi: 10.1002/ece3.72841 (PMC12862279; doi:10.1002/ece3.72841)

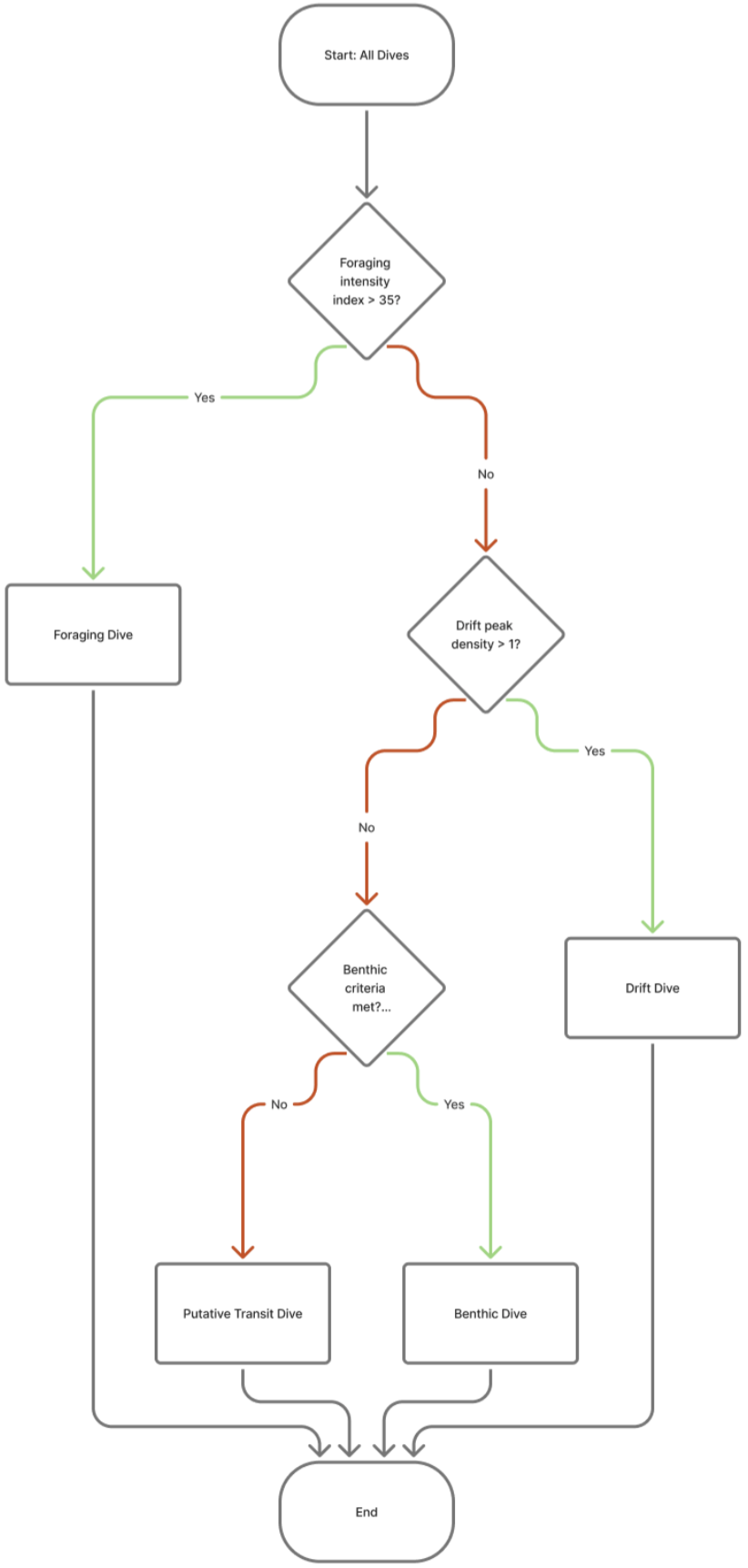


Supplemental Materials Figure 1: Decision tree used for dive typing.

Supplement: Supplementary file 1 — Figure S1: ece372841‐sup‐0001‐FigureS1.docx. [file ECE3-16-e72841-s001.docx]
